# Late Outcomes of Permanent Pacemaker Implantation After TAVR: Meta-analysis of Reconstructed Time-to-Event Data

**DOI:** 10.1016/j.jscai.2022.100434

**Published:** 2022-08-13

**Authors:** Michel Pompeu Sá, Xander Jacquemyn, Tian Sun, Jef Van den Eynde, Panagiotis Tasoudis, Ozgun Erten, Serge Sicouri, Gianluca Torregrossa, Marie-Annick Clavel, Philippe Pibarot, Basel Ramlawi

**Affiliations:** a Department of Cardiothoracic Surgery, Lankenau Heart Institute, Lankenau Medical Center, Main Line Health, Wynnewood, Pennsylvania; b Department of Cardiothoracic Surgery Research, Lankenau Institute for Medical Research, Wynnewood, Pennsylvania; c Department of Cardiovascular Sciences, KU Leuven, Leuven, Belgium; d Centre de Recherche de l’Institut Universitaire de Cardiologie et de Pneumologie de Québec, Québec City, Québec, Canada; e Department of Medicine, Faculty of Medicine, Université Laval, Québec City, Québec, Canada

**Keywords:** aortic valve, cardiac surgical procedures, cardiovascular surgical procedures, heart valve prosthesis implantation, meta-analysis, transcatheter aortic valve replacement

## Abstract

**Background:**

Permanent pacemaker implantation (PPI) after transcatheter aortic valve replacement (TAVR) is relatively frequent, and its impact on outcomes during follow-up remains a matter of discussion. Previous meta-analyses have yielded conflicting results.

**Methods:**

To compare late outcomes in patients after TAVR with and without PPI, PubMed/MEDLINE, Embase, and Google Scholar were searched for studies that reported rates of mortality/survival, rehospitalization for heart failure (HF), stroke, and/or endocarditis accompanied by at least 1 Kaplan-Meier curve for any of these outcomes. We adopted a 2-stage approach to reconstruct individual patient data on the basis of the published Kaplan-Meier graphs.

**Results:**

Twenty-eight studies with Kaplan-Meier curves met our eligibility criteria and included a total of 50,282 patients (7232 who underwent PPI and 42,959 who did not undergo PPI). Patients who underwent PPI after TAVR had a significantly higher risk of mortality (hazard ratio [HR], 1.21; 95% CI, 1.14-1.28; *P* < .001) and HF-related rehospitalization (HR, 1.30; 95% CI, 1.17-1.45; *P* < .001) over time. We did not observe statistically significant differences in the incidence of stroke (HR, 1.07; 95% CI, 0.55-2.08; *P* = .849) and endocarditis (HR, 0.98; 95% CI, 0.61-1.57; *P* = .925) during follow-up.

**Conclusions:**

Patients who undergo PPI after TAVR experience higher risk of mortality and HF-related rehospitalization over time. These findings provide support for the implementation of procedural strategies to prevent heart conduction disorder and, thus, avoid PPI at the time of TAVR.

## Highlights

• Pacemaker implantation after transcatheter aortic valve replacement is associated with higher risk of all-cause mortality
• Pacemaker implantation after transcatheter aortic valve replacement carries a higher risk of rehospitalization for heart failure
• There was no higher risk of stroke or endocarditis associated with permanent pacemaker implantation after transcatheter aortic valve replacement
• Efforts to prevent conduction abnormalities requiring permanent pacemaker implantation after transcatheter aortic valve replacement should be made

## Central Illustration

**Figure:**
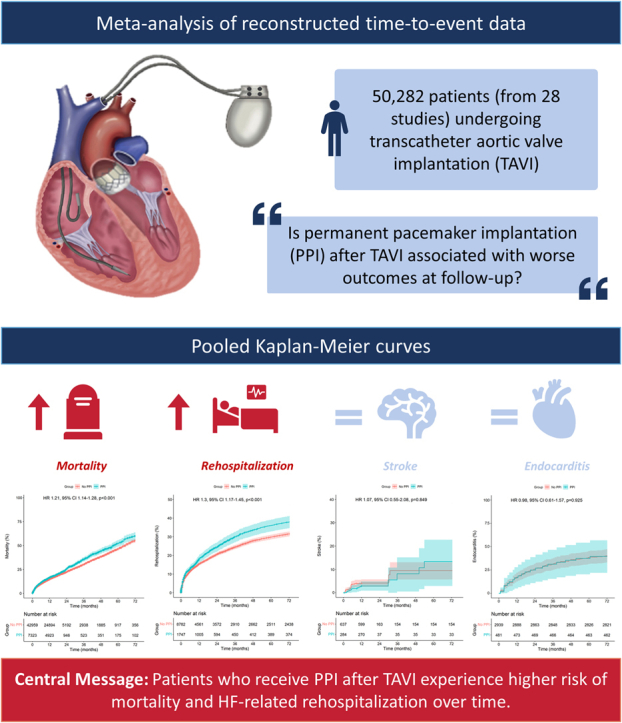

## Introduction

Over the last 20 years, transcatheter aortic valve replacement (TAVR) has become the treatment of choice for patients at prohibitive/high or intermediate surgical risk,^1^ and, more recently, new clinical trials have demonstrated its benefit (or noninferiority) in patients at low surgical risk.^2^^,^^3^ Among several post-TAVR complications, conduction abnormalities requiring permanent pacemaker implantation (PPI) are considered relatively common and are associated with clinical and technical factors.^4^

When compared with surgical aortic valve replacement, the occurrence of post-TAVR PPI has been reported to be higher.^5^ However, the impact of PPI after TAVR during follow-up remains a matter of discussion. Previous meta-analyses have yielded conflicting results.^6^, ^7^, ^8^, ^9^

Despite the good quality of these meta-analyses, most authors pool their data using mostly random-effects models to produce incidence rate ratios, odds ratios, or risk ratios as summary measures. Time-to-event outcomes are not easily incorporated into traditional meta-analyses. Researchers have resorted to pooling median survival times, incidence rate ratios, or event rates estimated from survival estimates at given time points or made direct estimates of hazard ratios (HRs) across the studies. All these approaches have been shown to be limiting and unsatisfactory because they do not allow the production of pooled Kaplan-Meier curves and fail to recognize some of the central tenets of survival analysis, such as censoring and the proportional hazards assumption.^10^ In response to the inconsistent reporting that resulted from these diverging approaches, the “curve approach” has emerged as the current standard for meta-analysis of aggregated time-to-event data.^11^ This approach reconstructs individual patient data (IPD) on the basis of published Kaplan-Meier graphs from the included studies.

The objective of this study was to evaluate the association between PPI after TAVR and the risk of all-cause mortality, rehospitalization for heart failure (HF), stroke, and endocarditis during follow-up. To address this objective, we performed a pooled analysis of Kaplan-Meier–estimated IPD from studies comparing the outcomes of patients who underwent PPI after TAVR with those of patients who did not undergo PPI after TAVR.

## Materials and methods

### Eligibility criteria, databases, and search strategy

This study followed the Preferred Reporting Items for Systematic Reviews and Meta-analyses (PRISMA) reporting statement.^12^ Using the Population, Interventions, Comparison, Outcome, and Study design strategy, studies were included if the following criteria were fulfilled:

1. The population comprised adults with aortic valve disease, requiring TAVR.
2. There was a group of patients who underwent PPI after TAVR.
3. There was a group of patients who did not undergo PPI after TAVR.
4. The outcomes studied included survival/mortality, HF-related rehospitalization, stroke, and/or endocarditis, with at least 1 of these outcomes with Kaplan-Meier curves.
5. The study design was retrospective/prospective, randomized/nonrandomized, and mono/multicentric, with matched/unmatched populations.

The following sources were searched for articles meeting our inclusion criteria and published on or before December 31, 2021: PubMed/MEDLINE, Embase, Google Scholar, and the reference lists of relevant articles. We searched for the following terms: (“TAVI” OR “transcatheter aortic valve implantation” OR “transcatheter aortic valve replacement” OR “TAVR”) AND (“PPI” OR “permanent pacemaker implantation” OR “artificial pacemakers” OR “artificial cardiac pacemaker”). Studies were selected by 2 independent reviewers (T.S. and J.V.E.). When there was disagreement, a third reviewer (M.P.S.) made the decision to include or exclude the study.

### Assessment of risk of bias

The Risk of Bias in Non-Randomized Studies of Interventions tool was systematically used to assess the included studies for risk of bias.^13^ Two independent reviewers (P.T. and O.E.) assessed the risk for bias. When there was a disagreement, a third reviewer (M.P.S.) checked the data and made the final decision.

### Statistical analysis

We used the 2-stage approach as described by Liu et al^14^ based on the R package “IPDfromKM” (version 0.1.10). In the first stage, raw data coordinates (time and survival probability) were extracted from each treatment arm in each of the Kaplan-Meier curves. In the second stage, the data coordinates were processed on the basis of the raw data coordinates from the first stage in conjunction with the numbers at risk at given time points, and IPD were reconstructed. Finally, the reconstructed IPD from all the studies were merged to create the study data set. The cumulative incidence of each outcome at follow-up in both arms (with and without PPI after TAVR) was visually assessed using Kaplan-Meier estimates with the R packages “survival” (version 3.2-13) and “survminer” (version 0.4.9). HRs with 95% confidence intervals (CIs) for the difference between both the treatment arms were calculated using a Cox regression model with the R package “coxphw” (version 4.0.2). In this study, an HR of >1 (with a *P* value of <.05) indicated a higher risk for a certain outcome after TAVR. We accounted for statistical heterogeneity among the included studies with a random intercept parameter. The proportionality of the hazards of each Cox model was checked with the Grambsch-Therneau test and diagnostic plots based on Schoenfeld residuals. One-study-removed sensitivity analyses were planned to avoid excessive impact of disproportionally larger samples on pooled results. As an extra step, when statistically significant differences for any outcomes were found, HRs and 95% CI values were derived from each Kaplan-Meier curve and pooled using a random-effects model. All analyses were completed with R Statistical Software version 4.1.1 (Foundation for Statistical Computing).

## Results

### Study selection and characteristics

**Figure 1:**
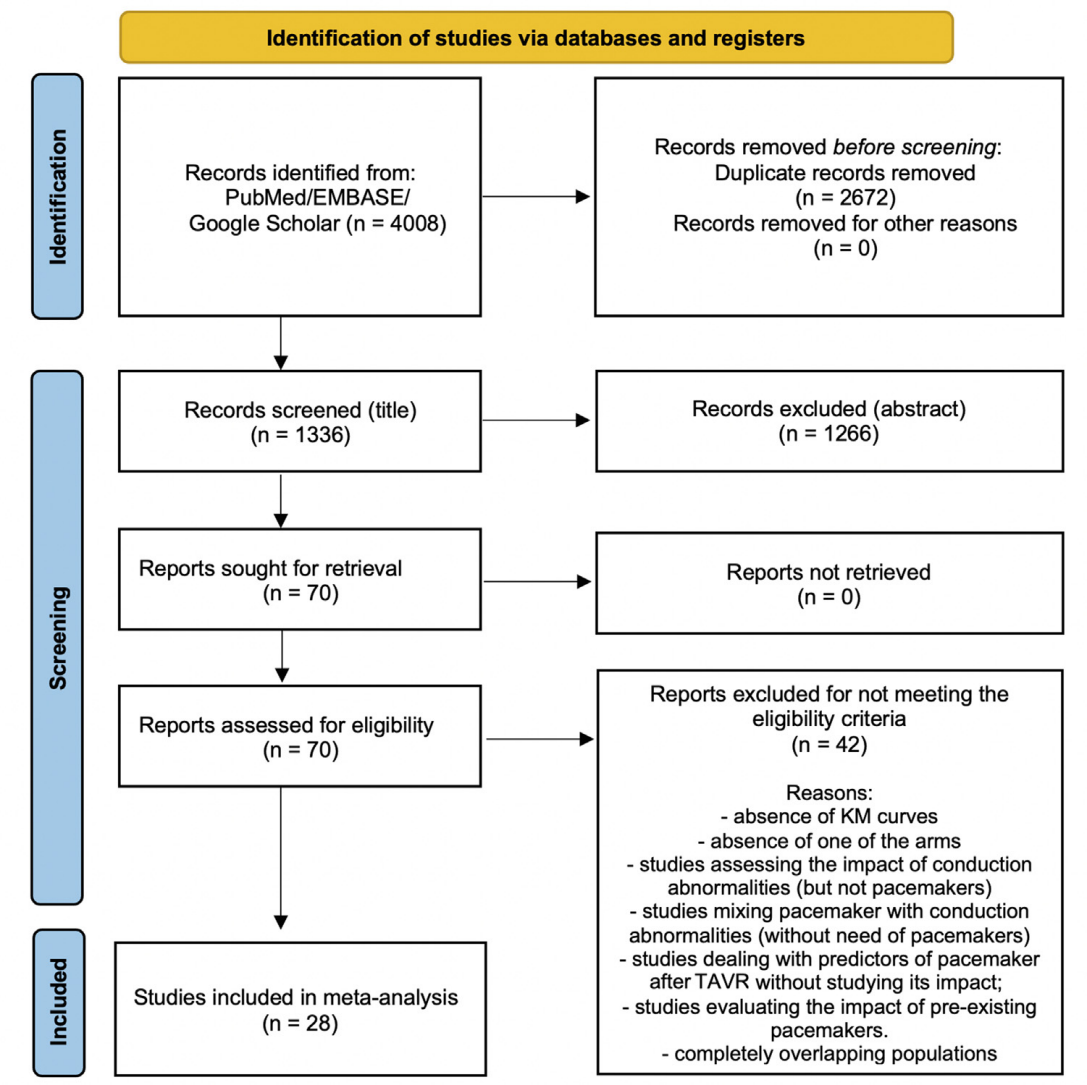
**Flow diagram of studies included in data search**. A total of 4008 records were identified, 70 full-text articles were assessed for eligibility, and 28 articles were included in the study. KM, Kaplan-Meier; TAVR, transcatheter aortic valve replacement.

**Figure 2:**
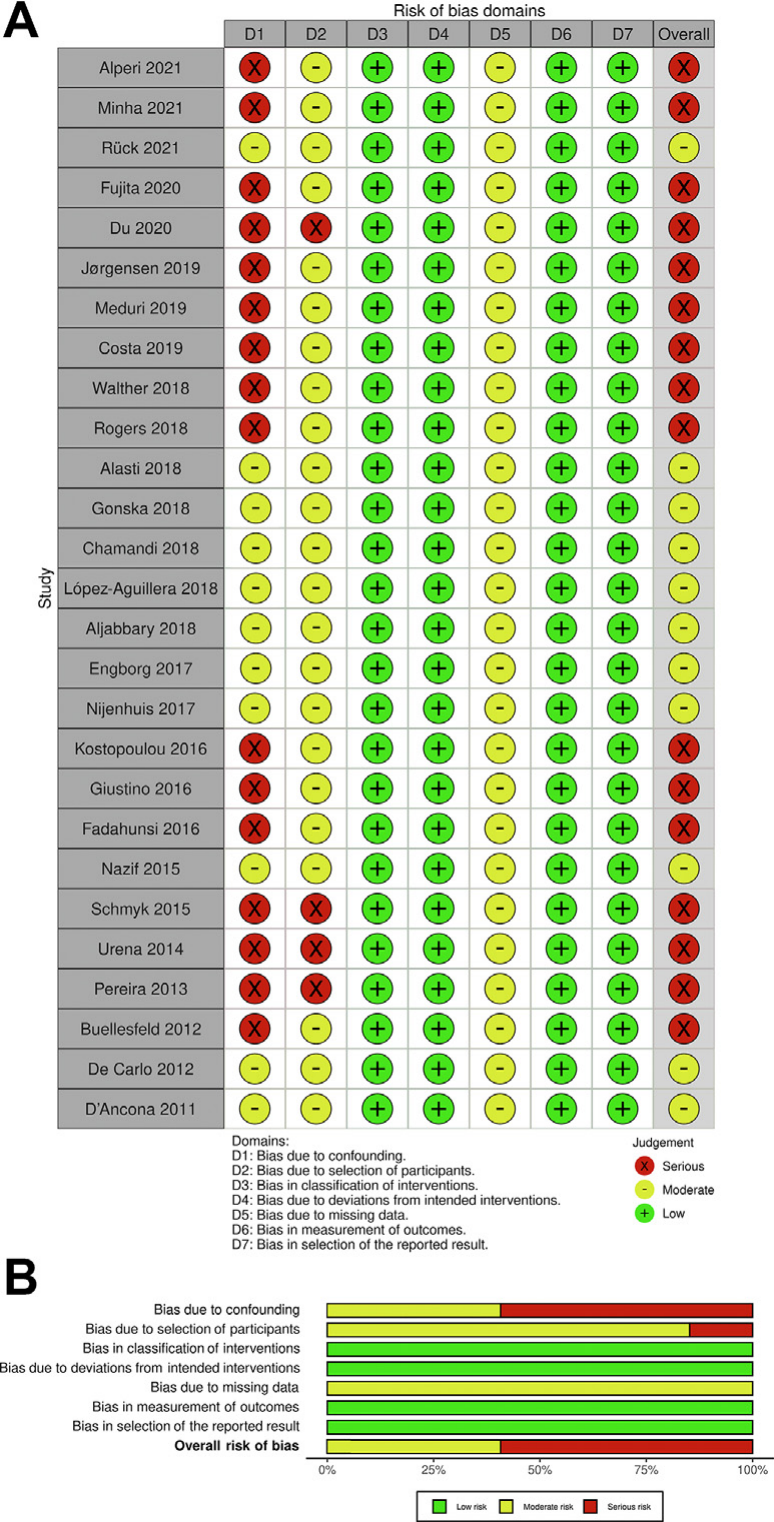
**Risk of bias summary.** The Risk of Bias in Non-Randomized Studies of Interventions tool with (**A**) traffic lights and (**B**) summary plots.

After excluding duplicates and noneligible studies, 28 studies^15^, ^16^, ^17^, ^18^, ^19^, ^20^, ^21^, ^22^, ^23^, ^24^, ^25^, ^26^, ^27^, ^28^, ^29^, ^30^, ^31^, ^32^, ^33^, ^34^, ^35^, ^36^, ^37^, ^38^, ^39^, ^40^, ^41^, ^42^ met our eligibility criteria (Figure 1). Almost all the studies were nonrandomized and observational, whereas 14 studies were multicentric and 17 studies included prospective populations (Table 1). A total of 50,282 patients were included in the original studies (7232 patients who underwent PPI after TAVR and 42,959 patients who did not undergo PPI after TAVR). The overall incidence of PPI after TAVR was 14.4% and ranged from 6.4% to 32.8%. All the studies included in our meta-analysis excluded patients with preexisting pacemakers. Table 2 shows the characteristics of the patients included in the studies. Figure 2 shows qualitative assessment of the studies with the Risk of Bias in Non-Randomized Studies of Interventions tool. We have some concerns related to missing data and confounding factors; for example, very few studies reported the type of pacemaker implanted and its pacing mode (Supplemental Table S1), presence of left ventricle outflow tract calcification, prosthesis-patient mismatch, or paravalvular leakage after TAVR (all factors which may affect the outcomes).

**Table 1:** Studies included.

Reference, year	Design	Total patients (N)	PPI n/N (%)	BEV (%)	SEV (%)	MEV (%)
Alperi et al,^15^ 2021	O, P, M	1987	128/1987 (6.4)	40.5	59.5	0
Minha et al,^16^ 2021	O, R, M	1377	276/1377 (20.0)	36.2	63.8	0
Rück et al,^17^ 2021	O, R, M	3420	481/3420 (14.1)	38.4	NR	NR
Fujita et al,^18^ 2020	O, P, M	20,872	3459/20,872 (16.6)	53.7	37.5	NR
Du et al,^19^ 2020	O, R, S	256	38/256 (14.8)	0	100	0
Jørgensen et al,^20^ 2019	O, P, S	816	132/816 (16.2)	9.4	82.6	8
Meduri et al,^21^ 2019	P, M	864	245/864 (28.4)	NR	NR	NR
Costa et al,^22^ 2019	O, P, S	1116	145/1116 (13)	27.2	72.5	NR
Walther et al,^23^ 2018	O, P, M	196	33/196 (16.7)	0	100	0
Rogers et al,^24^ 2018	O, R, S	614	145/614 (23.6)	77.8	22.1	0
Alasti et al,^25^ 2018	O, P, S	152	38/152 (25.0)	0	0	100
Gonska et al,^26^ 2018	O, R, S	612	168/612 (27.5)	58.8	4.4	36.8
Chamandi et al,^27^ 2018	O, P, M	1629	322/1629 (19.8)	43.8	53.9	NR
López-Aguillera et al,^28^ 2018	O, P, S	217	39/217 (17.9)	NR	NR	NR
Aljabbary et al,^29^ 2018	O, R, M	1263	186/1263 (14.7)	NR	NR	NR
Engborg et al,^30^ 2017	O, P, S	128	41/128 (32)	21.9	78.1	0
Nijenhuis et al,^31^ 2017	O, R, S	155	37/155 (24)	NR	NR	NR
Kostopoulou et al,^32^ 2016	O, P, S	30	8/30 (26.7)	NR	NR	NR
Giustino et al,^33^ 2016	O, R, M	947	145/947 (13.2)	47.9	52.1	0
Fadahunsi et al,^34^ 2016	O, R, M	9785	651/9785 (6.7)	88.8	11.2	0
Nazif et al,^35^ 2015	RCT, M	1973	173/1973 (8.8)	NR	NR	NR
Mouillet et al,^36^ 2015	O, P, M	833	252/833 (30.3)	0	100	0
Schymik et al,^37^ 2015	O, P, S	634	69/634 (10.8)	80.8	19.2	0
Urena et al,^38^ 2014	O, P, M	1556	239/1556 (15.4)	55	45	0
Pereira et al,^39^ 2013	O, R, S	58	19/58 (32.8)	NR	NR	NR
Buellesfeld et al,^40^ 2012	O, P, M	305	98/305 (32.1)	10.5	89.5	0
De Carlo et al,^41^ 2012	O, P, M	275	66/275 (24)	0	100	0
D’Ancona et al,^42^ 2011	O, P, S	322	20/322 (6.2)	100	0	0

BEV, balloon-expandable valve; M, multicentric; MEV, mechanically expandable valve; NR, nonreported; O, observational; P, prospective; PPI, permanent pacemaker implantation; R, retrospective; RCT, randomized controlled trial; S, single-center study; SEV, self-expandable valve.

**Table 2:** Characteristics of the patients included in the studies.

Reference, year	Age (y), mean PPI/no PPI	Female sex (%) PPI/no PPI	Diabetes mellitus (%) PPI/no PPI	CAD (%) PPI/no PPI	AF (%) PPI/no PPI	STS score (mean/median) PPI/no PPI	LVEF (%) (mean) PPI/no PPI
Alperi et al,^15^ 2021	80.2/77.4^a^	46.5/43.6	26.4/25.4	NR	25.4/20.5	6.0/6.1	54.2/53.0
Minha et al,^16^ 2021	NR	NR	NR	NR	NR	NR	NR
Rück et al,^17^ 2021	81.7/81.2	43.9/51.4	32.8/28.2	30.4/29.8	43.7/39.6	NR	NR
Fujita et al,^18^ 2020	NR	NR	NR	NR	NR	NR	NR
Du et al,^19^ 2020	76.6/75.5	39.5/42.7	21.5/21.6	21.1/8.7	18.4/16.1	6.5/7.2	55.6/52.6
Jørgensen et al,^20^ 2019	80/81	54.5/48.7	21.2/20.2	39.4/51.0	39.4/32.5	3.4/3.3	60/55
Meduri et al,^21^ 2019	83/82	50/51	34/30	72/70	4.6/3.1	6.8/6.5	NR
Costa et al,^22^ 2019	82/82	55.9/58.6	23.4/20.1	23.4/20.1	17.2/16.2	4.4/4.4	54.3/53.2
Walther et al,^23^ 2018	82.1/83.3	69.7/78.8	42.4/26.7	66.7/58.2	30.3/20.6	6.1/5.7	54.9/60.7^a^
Rogers et al,^24^ 2018	82.3/82.7	53.2/53.9	35.8/31.8	68.4/72.5	47.8/33.4^a^	8.0/8.8^a^	55.1/53.8
Alasti et al,^25^ 2018	84.6/83.4	41.0/58.0	8.0/22.0	21.0/12.0	18.0/30.0	NR	61.1/58.8
Gonska et al,^26^ 2018	81.1/80.1	47.6/55.0	25.6/31.5	63.5/60.8	39.3/34.7	6.7/6.6	58.0/57.1
Chamandi et al,^27^ 2018	82/81	43.8/41.2	32.3/32.5	44.1/42.0	21.2/21.4	7.4/6.9	57/56
López-Aguillera et al,^28^ 2018	78/78	41/55	33.3/28.6	25.6/34.2	23.0/25.3	10.7/11.6	62/58
Aljabbary et al,^29^ 2018	82.4/82.4	46.7/47.1	45.4/47.4	74.5/72.0	24.3/26.2	8.9/8.4	NR
Engborg et al,^30^ 2017	82.1/79.9	42/61^a^	17/18	22/15	32/22	15.5/17.9^b^	53.4/48.9
Nijenhuis et al,^31^ 2017	81/80	41/53	32/22	68/54	57/34^a^	7/5	59/59
Kostopoulou et al,^32^ 2016	78/82	60/34	NR	NR	10/17	NR	51/49
Giustino et al,^33^ 2016	82.2/80.8	40.2/50.6	28.8/28.6	24.4/30.5	24.7/22.1	8.1/8.7	52.3/52.2
Fadahunsi et al,^34^ 2016	84/84	47.8/53.1^a^	36.4/34.7	38.4/34.8	39.6/36.9	7.3/6.7^a^	57/58
Nazif et al,^35^ 2015	84.8/84.2	53.8/51.4	35.8/36.3	80.9/75.8	22.8/23.6	11.5/11.3	53.5/53.9
Mouillet et al,^36^ 2015	81.6/82.4	36.1/43.5^a^	25.5/24.8	48.2/45.8	30.9/26.9	12.2/14.9^a^	53.8/52.7
Schymik et al,^37^ 2015	NR	NR	NR	NR	NR	NR	NR
Urena et al,^38^ 2014	81/80	53.6/52.2	28.0/31.7	46.9/58.1^a^	25.9/28.2	7.2/7.7	56/55
Pereira et al,^39^ 2013	NR	NR	NR	NR	NR	NR	NR
Buellesfeld et al,^40^ 2012	82.5/82.6	53.1/61.4	28.6/23.7	58.1/52.7	20.4/22/7	27.7/22.7^a^^,^^b^	49.2/52.2
De Carlo et al,^41^ 2012	82.3/81.9	43.9/56.4	NR	NR	NR	23.1/22.1^b^	52.1/51.7
D’Ancona et al,^42^ 2011	82.4/79.1	60/67	20/25	30/62^a^	25/29	13.4/18.8^a^	50.5/50.5

AF, atrial fibrillation; CAD, coronary artery disease; LVEF, left ventricular ejection fraction; NR, nonreported; PPI, permanent pacemaker implantation; STS, Society of Thoracic Surgeons.

a *P* < .05.

b EuroSCORE.

### Analysis of adverse events

**Figure 3:**
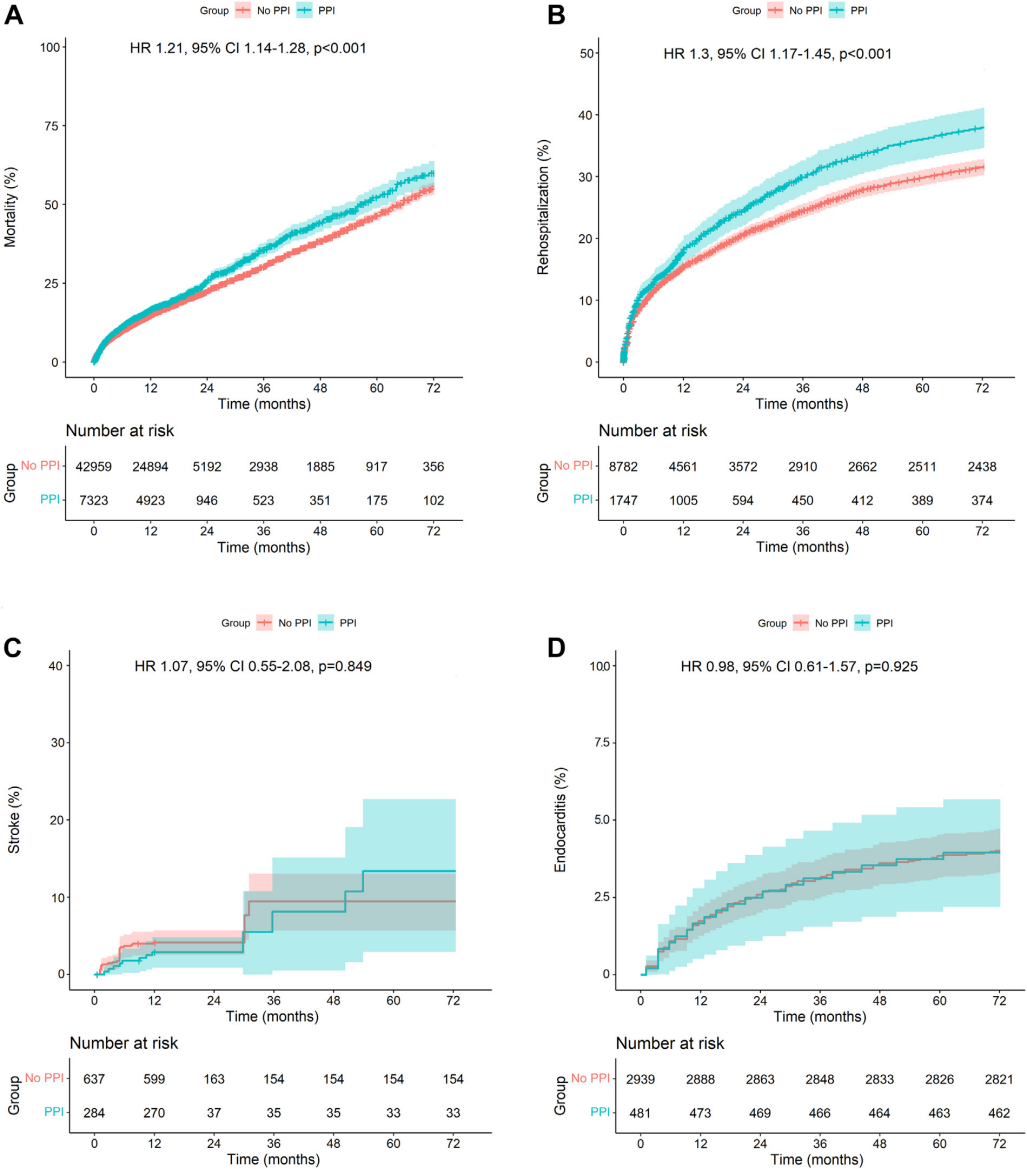
**Pooled Kaplan-Meier curves showing the cumulative risk of all-cause mortality, rehospitalization, stroke, and endocarditis after transcatheter aortic valve replacement with and without PPI.** (**A** and **B**) Shows the impact of PPI on mortality and rehospitalization, whereas (**C** and **D**) does not show any statistically significant differences. HR, hazard ratio; PPI, permanent pacemaker implantation.

**Supplementary Figure 1:**
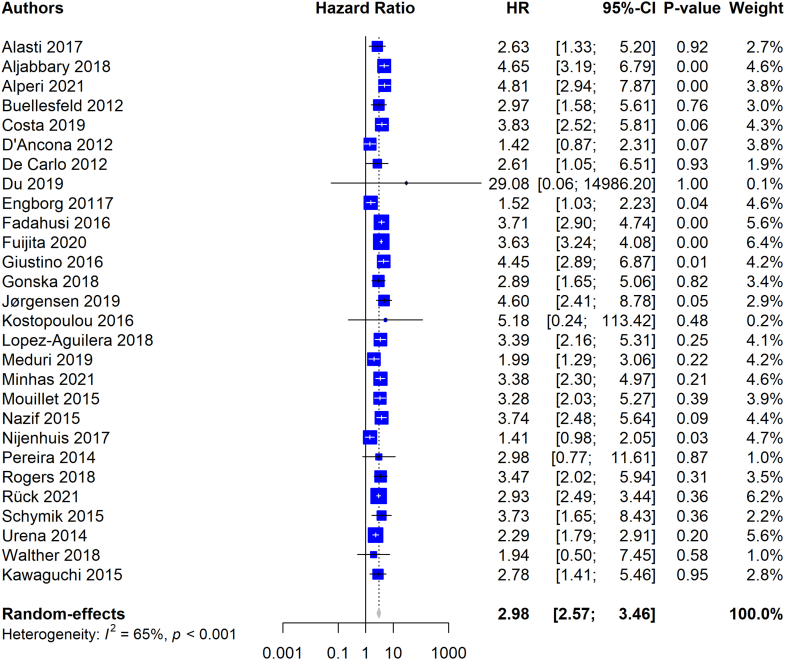


Figure 3A depicts the pooled Kaplan-Meier curve for the cumulative risk of all-cause mortality in all the included studies reporting PPI. The data of 50,282 patients (PPI, 7323 patients; no PPI, 42,959 patients) from 28 studies were pooled. PPI after TAVR was associated with a statistically significantly higher risk of mortality during follow-up (HR, 1.30; 95% CI, 1.17-1.45; *P* < .001). Pooling the data using a random-effects model also revealed higher risk of all-cause death in the group with PPI (Supplemental Figure S1).

**Supplementary Figure 2:**
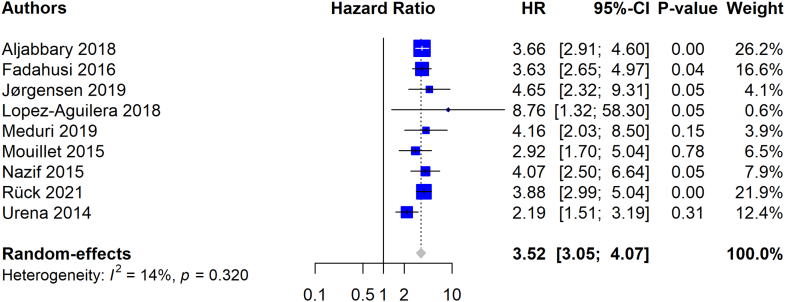


Figure 3B depicts the pooled Kaplan-Meier curve for the cumulative risk of HF-related rehospitalization. The data of 20,314 patients (PPI, 2398 patients; no PPI, 17,916 patients) from 9 studies were pooled. PPI after TAVR was associated with a statistically significantly higher risk of HF-related rehospitalization during follow-up (HR, 1.30; 95% CI, 1.17-1.45; *P* < .001). Pooling the data using a random-effects model also revealed higher risk of HF-related hospitalization in the group with PPI (Supplemental Figure S2).

Figure 3C depicts the pooled Kaplan-Meier curve for the cumulative risk of stroke. The data of 921 patients (PPI, 284 patients; no PPI, 637 patients) from 2 studies were pooled. Patients who underwent PPI had a risk of stroke comparable with that in patients who did not undergo PPI (HR, 1.07; 95% CI, 0.55-2.08; *P* = .849).

Figure 3D depicts the Kaplan-Meier curve for the cumulative risk of endocarditis. The data of 3420 patients (PPI, 481 patients; no PPI, 2939 patients) were reconstructed. Patients who underwent PPI had a risk of endocarditis comparable with that in patients who did not undergo PPI (HR, 0.98; 95% CI, 0.61-1.57; *P* = .925).

### Sensitivity analysis

**Figure 4:**
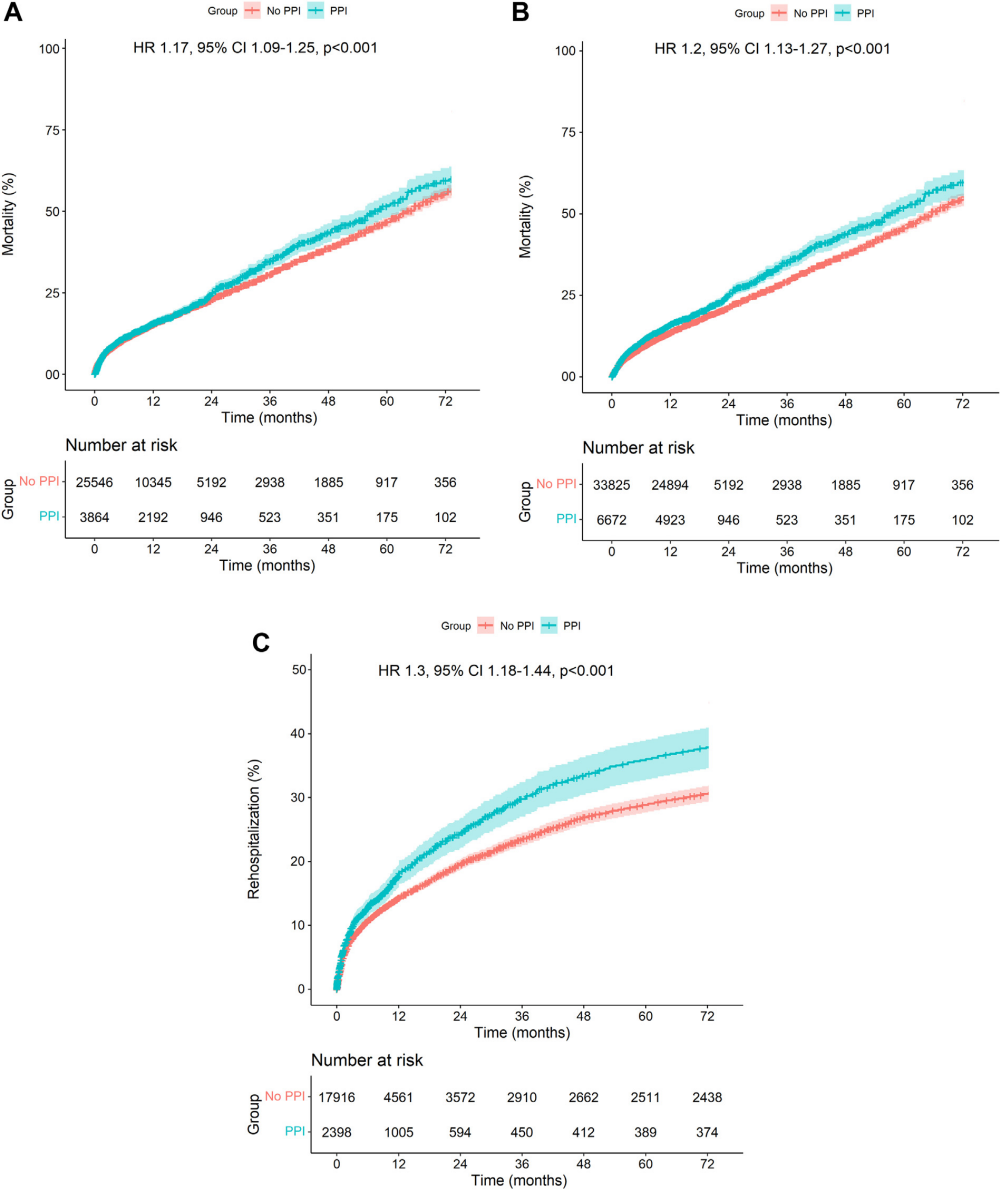
**Sensitivity analysis (1-study-removed analysis).** (**A-C**) All Kaplan-Meier curves shows the consistent impact of PPI on mortality and rehospitalization despite the removal of some studies from the analyses. HR, hazard ratio; PPI, permanent pacemaker implantation.

Because Fujita et al^18^ and Fadahunsi et al.^34^ contributed a disproportionately large number of patients to the mortality analyses (n = 20,872 [41.5%] and n = 9785 [19.5%], respectively), a sensitivity analysis was conducted, excluding these studies subsequently. Exclusion of the former left the data of 29,410 patients (no PPI, 25,546 patients; PPI, 3864 patients) from the remaining studies available for analysis of mortality. Exclusion of the latter left the data of 40,497 patients (no PPI, 33,825 patients; PPI, 6672 patients) from the remaining studies available for analysis of mortality. As depicted in Figure 4A and B, the sensitivity analyses confirmed the association of PPI with higher risk of mortality (HR, 1.17; 95% CI, 1.09-1.25; *P* < .001 and HR, 1.20; 95% CI, 1.13-1.27; *P* < .001).

In the analysis of HF-related rehospitalization, Fadahunsi et al^34^ also contributed a disproportionately large number of patients (n = 9785, 48.2%). Thus, a sensitivity analysis was conducted. Exclusion of this study left the data of 10,529 patients (no PPI, 8782 patients; PPI, 1747 patients) from the remaining studies available for analysis of rehospitalization. As depicted in Figure 4C, the sensitivity analysis confirmed the association of PPI with higher risk of rehospitalization (HR, 1.30; 95% CI, 1.18-1.44; *P* < .001).

## Discussion

To our knowledge, this is the first meta-analysis of reconstructed time-to-event data comparing late outcomes of TAVR with and without PPI. Our study complements and expands on the most recent meta-analysis published by Zito et al^6^ by doing the following:

1. Adding new recently published studies.
2. Adding pooled Kaplan-Meier curves to be visualized with HRs (instead of risk ratios) with longer follow-up for all-cause mortality and HF-related rehospitalization, respecting the principles of survival analysis, such as censoring and the proportional hazards assumption.
3. Confirming the higher risk of all-cause mortality and HF-related rehospitalization in those who underwent PPI after TAVR.
4. Adding endocarditis as an outcome of interest and showing the absence of any additional risk regarding this outcome.

The main finding of this study is that PPI after TAVR is associated with increased risk of all-cause death and HF-related hospitalization (Central Illustration). Ventricular dyssynchrony associated with right ventricular (RV) pacing^43^ may contribute to the higher risk of all-cause death and HF-related rehospitalization among patients who underwent PPI after TAVR. In this population, absence of mechanical synchrony after afterload relief (induced by preprocedural left ventricle hypertrophy) combined with ventricular dyssychrony (electrically induced by RV pacing) may not only hamper the postprocedural normalization of cardiac function but also induce further decline in function, which may account for the adverse clinical outcomes associated with chronic RV pacing after TAVR.^43^ Further studies are needed to determine whether single-chamber/dual-chamber pacemakers and pacing mode may yield different clinical outcomes during follow-up.

**Central Illustration:**
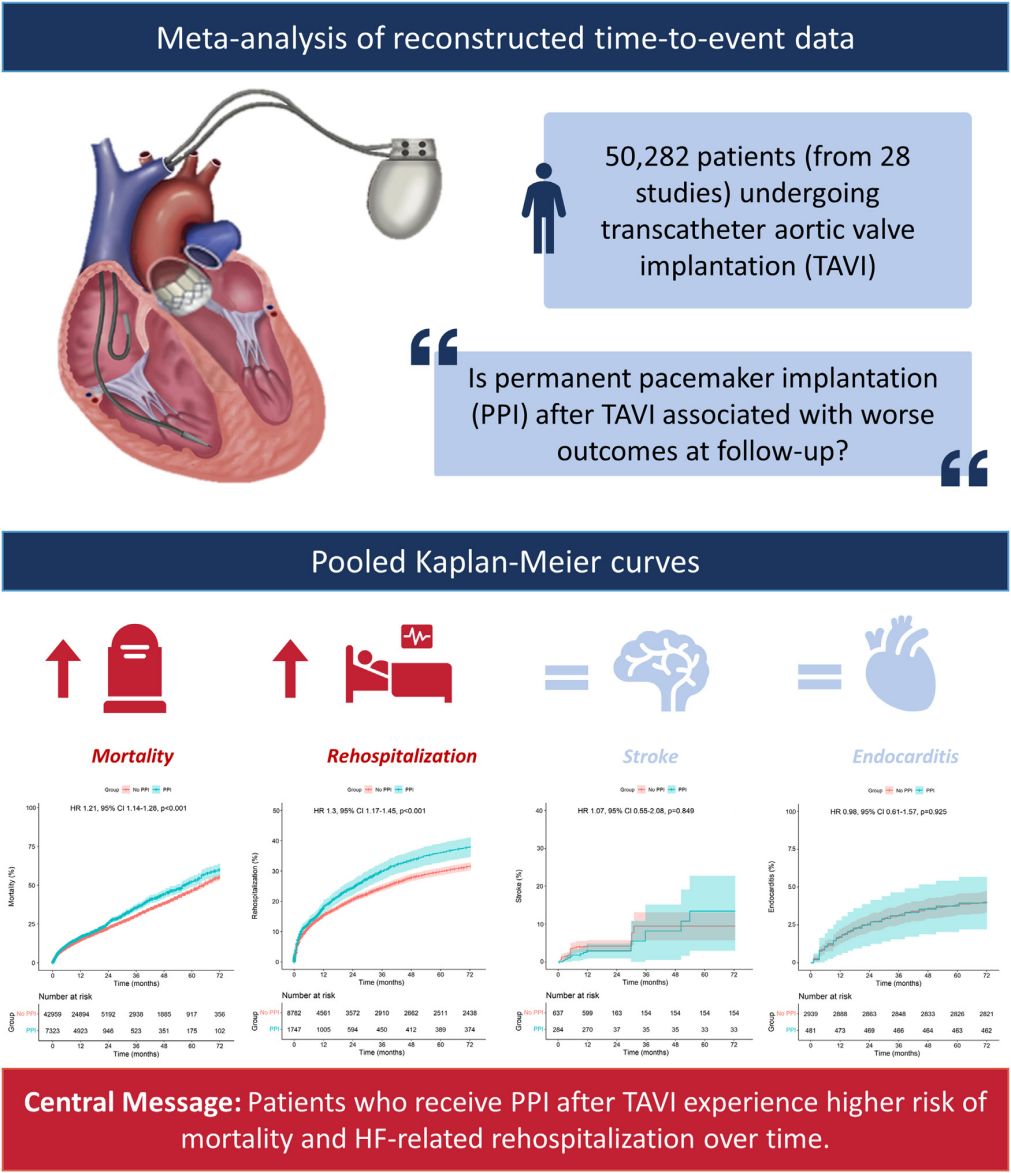
Impact of permanent pacemaker implantation after transcatheter aortic valve implantation. HF, heart failure; HR, hazard ratio; PPI, permanent pacemaker implantation; TAVR, transcatheter aortic valve replacement.

One of the limitations of our study is the lack of reporting of types of pacemaker and pacing mode in the original studies, which precluded us from analyzing these aspects as modulating factors of the pooled results by means of sensitivity analyses. Furthermore, the lack of data regarding pacing rates and dependency precluded us from evaluating the impact of these variables on outcomes. Additionally, the lack of real IPD (ours is Kaplan-Meier–derived IPD) regarding dependent and independent variables prevented us from establishing whether PPI after TAVR is an independent predictor of worse outcomes.

## Conclusions

Patients who undergo PPI after TAVR experience higher risk of mortality and HF-related rehospitalization over time. These findings provide support for the implementation of procedural strategies to prevent heart conduction disorder and, thus, avoid PPI at the time of TAVR.

## Supplementary material

Supplementary References
