# Supplementary material for: Late Outcomes of Permanent Pacemaker Implantation After TAVR: Meta-analysis of Reconstructed Time-to-Event Data
Source: J Soc Cardiovasc Angiogr Interv. 2022 Aug 13;1(5):100434. doi: 10.1016/j.jscai.2022.100434 (PMC11308092; doi:10.1016/j.jscai.2022.100434)
Supplement: Supplementary References [file mmc1.docx]

**Supplemental Reference List – Studies Included**

1. Alperi A, Rodés-Cabau J, Simonato M, Tchetche D, Charbonnier G, Ribeiro HB, et al. Permanent Pacemaker Implantation Following Valve-in-Valve Transcatheter Aortic Valve Replacement: VIVID Registry. J Am Coll Cardiol. 2021;77(18):2263-2273.

2. Minha S, Yarkoni Y, Segev A, Finkelstein A, Danenberg H, Fefer P, et al. Comparison of permanent pacemaker implantation rate after first and second generation of transcatheter aortic valve implantation-A retrospective cohort study. Catheter Cardiovasc Interv. 2021;98(7):E990-E999.

3. Rück A, Saleh N, Glaser N. Outcomes Following Permanent Pacemaker Implantation After Transcatheter Aortic Valve Replacement: SWEDEHEART Observational Study. JACC Cardiovasc Interv. 2021;14(19):2173-2181.

4. Fujita B, Schmidt T, Bleiziffer S, Bauer T, Beckmann A, Bekeredjian R, et al.; GARY Executive Board. Impact of new pacemaker implantation following surgical and transcatheter aortic valve replacement on 1-year outcome. Eur J Cardiothorac Surg 2020;57:151–9.

5. Du F, Zhu Q, Jiang J, Chen H, Liu X, Wang J. Incidence and predictors of permanent pacemaker implantation in patients who underwent transcatheter aortic valve replacement: observation of a Chinese population. Cardiol 2020;145:27–34.

6. Jørgensen TH, Backer OD, Gerds TA, Bieliauskas G, Svendsen JH, Søndergaard L. Mortality and heart failure hospitalization in patients with conduction abnormalities after transcatheter aortic valve replacement. JACC Cardiovasc Interv 2019;12:52–61.

7. Meduri CU, Kereiakes DJ, Rajagopal V, Makkar RR, O’Hair D, Linke A et al. Pacemaker implantation and dependency after transcatheter aortic valve replacement in the REPRISE III Trial. J Am Heart Assoc 2019;8:e012594.

8. Costa G, Zappulla P, Barbanti M, Cirasa A, Todaro D, Rapisarda G et al. Pacemaker dependency after transcatheter aortic valve implantation: incidence, predictors and long-term outcomes. EuroIntervention 2019;15:875–83.

9. Walther T, Manoharan G, Linke A, Möllmann H, Holzhey D, Worthley SG et al. Incidence of new-onset left bundle branch block and predictors of new permanent pacemaker following transcatheter aortic valve replacement with the PorticoTM valve. Eur J Cardiothorac Surg 2018;54:467–74.

10. Rogers T, Devraj M, Thomaides A, Steinvil A, Lipinski MJ, Buchanan KD et al. Utility of invasive electrophysiology studies in patients with severe aortic stenosis undergoing transcatheter aortic valve implantation. Am J Cardiol 2018;121:1351–7.

11. Alasti M, Rashid H, Rangasamy K, Kotschet E, Adam D, Alison J et al. Long-term pacemaker dependency and impact of pacing on mortality following transcatheter aortic valve replacement with the LOTUS valve. Catheter Cardiovasc Interv 2018;92:777–82.

12. Gonska B, Keßler M, Wöhrle J, Rottbauer W, Seeger J. Influence of permanent pacemaker implantation after transcatheter aortic valve implantation with new-generation devices. Neth Heart J 2018;26:620–7.

13. Chamandi C, Barbanti M, Munoz-Garcia A, Latib A, Nombela-Franco L, Gutiérrez-Ibanez E, et al. Long-term outcomes in patients with new permanent pacemaker implantation following transcatheter aortic valve replacement. JACC Cardiovasc Interv 2018;11:301–10.

14. López-Aguilera J, Segura Saint-Gerons JM, Sánchez Fernández J, Mazuelos Bellido F, Pan Álvarez-Ossorio M, Suárez De Lezo J, et al. Long-term clinical impact of permanent cardiac pacing after transcatheter aortic valve implantation with the CoreValve prosthesis: a single center experience. Europace 2018;20:993–1000.

15. Aljabbary T, Qiu F, Masih S, Fang J, Elbaz-Greener G, Austin PC et al. Association of clinical and economic outcomes with permanent pacemaker implantation after transcatheter aortic valve replacement. JAMA Netw Open 2018;1:e180088.

16. Engborg J, Riechel-Sarup C, Gerke O, Mickley H, Sandgaard NC, Nissen H et al. Effect of permanent pacemaker on mortality after transcatheter aortic valve replacement. Scand Cardiovasc J 2017;51:40–6.

17. Nijenhuis VJ, Dijk VV, Chaldoupi SM, Balt JC, Berg JT. Severe conduction defects requiring permanent pacemaker implantation in patients with a new-onset left bundle branch block after transcatheter aortic valve implantation. Europace 2017;19:1015–21.

18. Kostopoulou A, Karyofillis P, Livanis E, Thomopoulou S, Stefopoulos C, Doudoumis K, et al. Permanent pacing after transcatheter aortic valve implantation of a CoreValve prosthesis as determined by electrocardiographic and electrophysiological predictors: a single-centre experience. Europace. 2016;18(1):131-7.

19. Giustino G, Boon RVD, Nicolas JD, Dumonteil N, Chieffo A, Jaegere PD et al. Impact of permanent pacemaker on mortality after transcatheter aortic valve implantation: the PRAGMATIC (Pooled Rotterdam-Milan-Toulouse in Collaboration) Pacemaker substudy. EuroIntervention 2016;12:1185–93.

20. Fadahunsi OO, Olowoyeye A, Ukaigwe A, Li Z, Vora AN, Vemulapalli S et al. Incidence, predictors, and outcomes of permanent pacemaker implantation following transcatheter aortic valve replacement: analysis from the U.S. Society of Thoracic Surgeons/American College of Cardiology TVT Registry. JACC Cardiovasc Interv 2016;9:2189–99.

21. Nazif TM, Dizon JM, Hahn RT, Xu K, Babaliaros V, Douglas PS et al. Predictors and clinical outcomes of permanent pacemaker implantation after transcatheter aortic valve replacement: the PARTNER (Placement of AoRtic TraNscathetER Valves) trial and registry. JACC Cardiovasc Interv 2015;8:60–9.

22. Mouillet G, Lellouche N, Yamamoto M, Oguri A, Dubois-Rande JL, Belle EV et al. Outcomes following pacemaker implantation after transcatheter aortic valve implantation with CoreValve® devices: results from the France 2 Registry. Catheter Cardiovasc Interv 2015;86:E158–66.

23. Schymik G, Tzamalis P, Bramlage P, Heimeshoff M, Würth A, Wondraschek R et al. Clinical impact of a new left bundle branch block following TAVI implantation: 1-year results of the TAVIK cohort. Clin Res Cardiol 2015;104:351–62.

24. Urena M, Webb JG, Tamburino C, Muñoz-García AJ, Cheema A, Dager AE et al. Permanent pacemaker implantation after transcatheter aortic valve implantation impact on late clinical outcomes and left ventricular function. Circulation 2014;129:1233–43.

25. Pereira E, Ferreira N, Caeiro D, Primo J, Adão L, Oliveira M et al. Transcatheter aortic valve implantation and requirements of pacing over time. Pacing Clin Electrophysiol 2013;36:559–69.

26. Buellesfeld L, Stortecky S, Heg D, Hausen S, Mueller R, Wenaweser P et al. Impact of permanent pacemaker implantation on clinical outcome among patients undergoing transcatheter aortic valve implantation. J Am Coll Cardiol 2012;60:493–501.

27. De Carlo M, Giannini C, Bedogni F, Klugmann S, Brambilla N, De Marco F, et al. Safety of a conservative strategy of permanent pacemaker implantation after transcatheter aortic CoreValve implantation. Am Heart J 2012;163:492–9.

28. D'Ancona G, Pasic M, Unbehaun A, Hetzer R. Permanent pacemaker implantation after transapical transcatheter aortic valve implantation. Interact Cardiovasc Thorac Surg 2011;13:373–6.
